# Ion Channel Expression and Characterization in Human Induced Pluripotent Stem Cell-Derived Cardiomyocytes

**DOI:** 10.1155/2018/6067096

**Published:** 2018-01-08

**Authors:** Zhihan Zhao, Huan Lan, Ibrahim El-Battrawy, Xin Li, Fanis Buljubasic, Katherine Sattler, Gökhan Yücel, Siegfried Lang, Malte Tiburcy, Wolfram-Hubertus Zimmermann, Lukas Cyganek, Jochen Utikal, Thomas Wieland, Martin Borggrefe, Xiao-Bo Zhou, Ibrahim Akin

**Affiliations:** ^1^First Department of Medicine, Faculty of Medicine, University Medical Centre Mannheim (UMM), University of Heidelberg, Mannheim, Germany; ^2^DZHK (German Center for Cardiovascular Research), Partner Sites, Heidelberg-Mannheim and Göttingen, Mannheim, Germany; ^3^Key Laboratory of Medical Electrophysiology of Ministry of Education, Institute of Cardiovascular Research, Southwest Medical University, Luzhou, Sichuan, China; ^4^Institute of Pharmacology and Toxicology, University of Göttingen, Göttingen, Germany; ^5^Stem Cell Unit, Clinic for Cardiology and Pneumology, University Medical Center Göttingen, Göttingen, Germany; ^6^Skin Cancer Unit, German Cancer Research Center (DKFZ), Heidelberg, Germany; ^7^Department of Dermatology, Venereology and Allergology, University Medical Center Mannheim, University of Heidelberg, Mannheim, Germany; ^8^Institute of Experimental and Clinical Pharmacology and Toxicology, Medical Faculty Mannheim, University of Heidelberg, Mannheim, Germany

## Abstract

**Background:**

Human induced pluripotent stem cell-derived cardiomyocytes (hiPSC-CMs) are providing new possibilities for the biological study, cell therapies, and drug discovery. However, the ion channel expression and functions as well as regulations in hiPSC-CMs still need to be fully characterized.

**Methods:**

Cardiomyocytes were derived from hiPS cells that were generated from two healthy donors. qPCR and patch clamp techniques were used for the study.

**Results:**

In addition to the reported ion channels, I_Na_, I_Ca-L_, I_Ca-T_, I_f_, I_NCX_, I_K1_, I_to_, I_Kr_, I_Ks_ I_KATP_, I_K-pH_, I_SK1–3_, and I_SK4_, we detected both the expression and currents of ACh-activated (KACh) and Na^+^-activated (KNa) K^+^, volume-regulated and calcium-activated (Cl-Ca) Cl^−^, and TRPV channels. All the detected ion currents except I_K1_, I_KACh_, I_SK_, I_KNa_, and TRPV1 currents contribute to AP duration. Isoprenaline increased I_Ca-L_, I_f_, and I_Ks_ but reduced I_Na_ and I_NCX_, without an effect on I_to_, I_K1_, I_SK1–3_, I_KATP_, I_Kr_, I_SK4_, I_KNa_, I_Cl-Ca_, and I_TRPV1_. Carbachol alone showed no effect on the tested ion channel currents.

**Conclusion:**

Our data demonstrate that most ion channels, which are present in healthy or diseased cardiomyocytes, exist in hiPSC-CMs. Some of them contribute to action potential performance and are regulated by adrenergic stimulation.

## 1. Introduction

Since the successful reprogramming of adult somatic cells to induced pluripotent stem (iPS) cells and generation of functional cardiomyocytes from human iPS cells (hiPSC-CMs) [1–4], hiPSC-CMs have been demonstrated to have electrophysiological and pharmacological properties including action potentials and responses to antiarrhythmic drugs which are similar to those of native cardiomyocytes [4–6]. In addition, emerging evidences indicate that the hiPSC-CMs derived from patients with genetic heart diseases recapitulated the phenotype of the disease [7–11]. For some functional studies, especially the electrophysiological studies, hiPSC-CMs have also important advantages over heterologous expression systems like Xenopus oocytes, human embryonic kidney (HEK) cells, and Chinese hamster ovary (CHO) cells lacking important constituents of cardiac ion channel macromolecular complexes that might be necessary for the normal electrophysiological characteristics. In addition, transgenic animals possess cardiac electrophysiological properties crucially different from those in humans. Therefore, it has been widely accepted that hiPSC-CMs are providing new opportunities for both mechanistic and therapeutic studies on some heart diseases. For both aims, efficient induction and purification of cardiomyocytes are required. High differentiation efficiencies of cardiomyocytes have been reported [12–14]. However, these methods still did not provide pure cardiomyocytes, and thus, more methods for improving the purity of iPSC-CMs including selecting markers have been established [15]. The stem cell-derived cardiomyocytes could be selected and purified by using Percoll gradient fractionation [16], drug selection of cells engineered with CM promoter-driven selectable markers [17], and fluorescence-activated cell sorting (FACS) selection of CMs by mitochondrial fluorescent dye labelling [18], by surface expression of signal regulatory protein [19], or by activated leukocyte cell adhesion molecule (ALCAM, also referred to as CD166) [20, 21].

Another requirement for the applications of hiPSC-CMs is that they should have functional properties similar to those of native human cardiomyocytes. However, a major obstacle for the application of hiPSC-CMs is the distinct differences between hiPSC-CMs and mature cardiomyocytes. For example, the hiPSC-CMs are smaller, displaying abnormal properties including spontaneous beating and heteromorphologic action potentials with reduced diastolic potential, compared with mature cardiomyocytes [22]. These are indicative of an immature phenotype of hiPSC-CMs. The reason for the immaturity is not clear. The abnormal electrical property probably results from the changed expression profile of ion channels. Ion channels in hiPSC-CMs have been till now only partially characterized. At present, it is known that the major cardiac ion channels such as Na^+^ (SCN5A), L-type Ca^2+^, I_to_, I_Kr_, I_Ks_, and I_f_ are functionally expressed in hiPSC-CMs, but I_K1_, which is important for resting potential in mature cardiomyocytes, is lacking or is expressed at a reduced level [9, 22]. Many other ion channels, which have been demonstrated to exist either in healthy or in diseased cardiomyocytes, have not been studied in hiPSC-CMs. Therefore, we designed this study to investigate the expression and function as well as the regulation of ion channels in hiPSC-CMs with a focus on those that have not yet been checked or have not been studied in detail.

## 2. Material and Methods

### 2.1. Ethics Statement

The skin biopsies from two healthy donors were obtained with written informed consent. The study procedures were approved by the Ethics Committee of Medical Faculty Mannheim, Heidelberg University (approval number: 2009-350N-MA), and carried out in accordance with the Helsinki Declaration of 1975, as revised in 1983.

### 2.2. Generation of Human iPS Cells

The human iPS cells (hiPSCs) were generated from primary human fibroblasts derived from skin biopsies of the two healthy donors. The hiPSC line from donor 1 (female, here abbreviated as D1) was generated using lentiviral particles carrying the transactivator rtTA and an inducible polycistronic cassette containing the reprogramming factors OCT4, SOX2, KLF4, and c-MYC as previously described [23, 24]. The hiPSC line from donor 2 (male, here abbreviated as D2) was generated using the integration-free episomal 4-in-1 CoMiP reprogramming plasmid (Addgene, #63726) with the reprogramming factors OCT4, KLF4, SOX2, and c-MYC and short hairpin RNA against p53, as described previously with modifications [25].

### 2.3. Generation of hiPSC-CMs

The hiPS cell lines were differentiated into cardiomyocytes as described in our previous studies [26, 27]. Cardiomyocytes 30 to 60 days after the onset of differentiation were dissociated from 24-well plates and used for PCR analysis or plated as single cells on Matrigel-coated 3.5 cm petri dishes for patch clamp measurements.

### 2.4. Polymerase Chain Reaction Assays

The preparation of total RNA using the RNeasy mini kit (Qiagen, Hilden, Germany) including DNAse treatment was performed following the protocol. The cDNA was amplified by qPCR on a Stratagene MX 3005P real-time cycler (Stratagene, USA) using a PCR mix with hot start Taq DNA polymerase and SYBR Green (Sibir Rox Hot Mastermix, BIORON, Germany; Cat number 119405) in the presence of sense and antisense primers (400 nM each). Relative mRNA expression level was calculated as the expression of the mRNA of the gene of interest relative to GAPDH in samples from treated or untreated (control) cells which was calculated by the ΔΔCT method, based on the threshold cycle (CT), as fold change = 2^−Δ(ΔCT)^, where ΔCT = CT_gene of interest_ − CT_GAPDH_ and Δ(ΔCT) = ΔCT_treated_ − ΔCT_control_ [20]. Results are shown as mean ± SEM from the measurements of 3 biological replicates and 2 technical replicates. The GenBank NCBI reference sequence number (RefSeq number) and catalog number of all the investigated genes are listed in Table S1.

### 2.5. Patch Clamp

The action potential (AP) and ion channel currents were measured by standard patch clamp recording techniques in the whole-cell configuration. All the experiments were carried out at room temperature (22–25°C). Different protocols were used for measuring different currents. To isolate one type of ion channel current from others, a specific channel blocker or solution was used. To minimize the effects of rundown of recorded currents on the results of experiments, we carefully monitored the time-dependent change of currents. Recordings were started when the current became stable, usually within 3 to 5 minutes.

APs were recorded in a current clamp mode. To elicit APs, a holding current of −40 pA was injected through the patch pipette into the cell and brief current pulses (2 ms, 1 nA) were applied at 1 Hz to trigger APs.

For measuring AP and K^+^ channel currents, the bath solution contained 130 mmol/L NaCl, 5.9 mmol/L KCl, 2.4 mmol/L CaCl_2_, 1.2 mmol/L MgCl_2_, 11 mmol/L glucose, and 10 mmol/L HEPES (pH 7.4 (NaOH)). For the transient outward K^+^ current (I_to_) measurements, 10 *μ*M nifedipine, 10 *μ*M TTX, and 1 *μ*M E-4031 were added in the bath solution to block I_Ca-L_, I_Na_, and I_Kr_, respectively. For slowly delayed rectifier (I_Ks_) measurements, 10 *μ*M nifedipine, 3 mM 4-AP, and 10 *μ*M TTX were added. The pipette solution contained 10 mM HEPES, 126 mM KCl, 6 mM NaCl, 1.2 mM MgCl_2_, 5 mM EGTA, 11 mM glucose, and 1 mM MgATP (pH 7.4 (KOH)). For measuring small and intermediate conductance calcium-activated potassium channel currents (I_SK1–3_ and I_SK4_), appropriate CaCl_2_ was added to get the free Ca^2+^ concentration of 0.5 *μ*M according to the calculation by the software MAXCHELATOR (http://web.stanford.edu/~cpatton/downloads.htm). For measurements of ATP-sensitive K^+^ channel currents (I_KATP_), the ATP-free pipette solution was used. For sodium-activated K^+^ current (I_KNa_) measurements, intracellular NaCl of 50 mM was used. For pH-sensitive current measurements, the pH value of extracellular solution was reduced to 6.0.

External solution for Cs^+^ currents through the rapidly delayed rectifier (I_Kr_) channels contained 140 mmol/L CsCl, 2 mmol/L MgCl_2_, 10 mmol/L HEPES, and 10 mmol/L glucose (pH = 7.4 (CsOH)). Pipette solution contained 140 mmol/L CsCl, 2 mmol/L MgCl_2_, 10 mmol/L HEPES, and 10 mmol/L EGTA (pH = 7.2 (CsOH)).

The bath solution for the transient receptor potential channel type V1–4 (TRPV1–4) current measurements contained 140 mmol/L NaCl, 5.4 mmol/L TEA-Cl, 2.0 mmol/L CaCl_2_, 1.0 mmol/L MgCl_2_, 10 mmol/L HEPES, and 10 mmol/L glucose (pH 7.4 (CsOH)). Na^+^ current was inactivated by a holding potential of 0 mV. Nifedipine and E-4031 were used to block I_Ca-L_ and I_Kr_, respectively. The pipette solution contained 135 mmol/L CsCl, 0.1 mmol/L CaCl_2_, 10 mmol/L EGTA, 1.0 mmol/L MgATP, 1.0 mmol/L MgCl_2_, 10 mmol/L HEPES, and 0.1 mmol/L NaGTP (pH 7.4 (CsOH)).

The bath solution for volume-regulated chloride channel currents (I_Cl-vol_) contained the following: for isotonic solution (290–300 mOsm/L), 100 mmol/L NaCl, 5 mmol/L CsCl, 1 mmol/L MgCl_2_, 1.5 mmol/L CaCl_2_, 10 mmol/L glucose, 10 mmol/L HEPES, and 70 mmol/L mannitol (pH 7.4 (NaOH)) and for hypotonic solution (220–230 mOsm/L), 100 mmol/L NaCl, 5 mmol/L CsCl, 1 mmol/L MgCl_2_, 1.5 mmol/L CaCl_2_, 10 mmol/L glucose, and 10 mmol/L HEPES (pH 7.4 (NaOH)). The pipette solution contained 40 mmol/L CsCl, 100 mmol/L Cs-aspartate, 1 mmol/L MgCl_2_, 1.93 mmol/L CaCl_2_, 5 mmol/L EGTA, 2 mmol/L ATP, 0.5 mmol/L GTP, and 5 mmol/L HEPES (pH 7.2 (CsOH)). For AP measurements, the isotonic solution contained 100 mmol/L NaCl, 5 mmol/L KCl, 1 mmol/L MgCl_2_, 1.5 mmol/L CaCl_2_, 10 mmol/L glucose, 10 mmol/L HEPES, and 70 mmol/L mannitol (pH 7.4 (NaOH)), and the hypotonic solution contained 100 mmol/L NaCl, 5 mmol/L KCl, 1 mmol/L MgCl_2_, 1.5 mmol/L CaCl_2_, 10 mmol/L glucose, and 10 mmol/L HEPES (pH 7.4 (NaOH)). The pipette solution contained 20 mmol/L KCl, 110 mmol/L K-aspartate, 1 mmol/L MgCl_2_, 0.5 mmol/L EGTA, 2 mmol/L ATP, 0.5 mmol/L GTP, and 10 mmol/L HEPES (pH 7.2 (KOH)).

The bath solution for the measurements of Ca^2+^-activated Cl^−^ currents (I_Cl-Ca_) contained 100 mmol/L NMG (N-methyl-D-glucamine), 20 mmol/L NaCl, 20 mmol/L TEA-Cl, 2 mmol/L CaCl_2_, 1 mmol/L MgCl_2_, 10 mmol/L glucose, 10 mmol/L HEPES, 5 mmol/L 4-AP (pH 7.4 (HCl)). The pipette solution contained 150 mmol/L NMG, 1 mmol/L MgCl_2_, 4 mmol/L Mg-ATP, 0.05 mmol/L BAPTA, and 10 mmol/L HEPES (pH 7.4 (40 HCl + glutamic acid)).

For peak sodium current (I_Na_) measurements, the bath solution contained 20 mmol/L NaCl, 110 mmol/L CsCl, 1.8 mmol/L CaCl_2_, 1 mmol/L MgCl_2_, 10 mmol/L HEPES, 10 mmol/L glucose, and 0.001 mmol/L nifedipine (pH 7.4 (CsOH)). Microelectrodes were filled with 10 mmol/L NaCl, 135 mmol/L CsCl, 2 mmol/l CaCl_2_, 3 mmol/L MgATP, 2 mmol/L TEA-Cl, 5 mmol/L EGTA, and 10 mmol/L HEPES (pH 7.2 (CsOH)).

The bath solution for L-type (I_Ca-L_) and T-type (I_Ca-T_) calcium channel current recordings contained 140 mmol/L TEA-Cl, 5 mmol/L CaCl_2_, 1 mmol/L MgCl_2_, 3 mmol/L E-4031, 10 mmol/L HEPES, 0.02 mmol/L TTX, and 3 mmol/L 4-AP (pH 7.4 (CsOH)). The pipette solution contained 6 mmol/L NaCl, 135 mmol/L CsCl, 2 mmol/L CaCl_2_, 3 mmol/L MgATP, 2 mmol/L TEA-Cl, 5 mmol/L EGTA, and 10 mmol/L HEPES (pH 7.2 (CsOH)).

The bath solution for Na^+^-Ca^2+^ exchanger current (I_NCX_) measurements contained 135 mmol/L NaCl, 10 mmol/L CsCl, 2 mmol/L CaCl_2_, 1 mmol/L MgCl_2_, 10 mmol/L HEPES, 10 mmol/L glucose, 0.01 mmol/L nifedipine, 0.1 mmol/L niflumic acid, 0.05 mmol/L lidocaine, and 0.02 mmol/L dihydroouabain (pH 7.4 (CsOH)). Microelectrodes were filled with 10 mmol/L NaOH, 150 mmol/L CsOH, 2 mmol/L CaCl_2_, 1 mmol/L MgCl_2_, 75 mmol/L aspartic acid, and 5 mmol/L EGTA (pH 7.2 (CsOH)).

### 2.6. Statistics

All the experiment data were analyzed using InStat© (GraphPad, San Diego, USA) and SigmaPlot 11 (Jandel Scientific). Parametric or nonparametric data were determined by analyzing the data with the Kolmogorov-Smirnov test. For nonparametric data, the Kruskal-Wallis test with Dunn's multiple comparison posttest was used. The paired *t*-test was used for comparison before and after application of a drug. Unpaired Student's *t*-test was used for comparisons of two independent groups with normal distribution. All the data are shown as mean ± SEM. *p* < 0.05 (two-tailed) was considered significant.

## 3. Results

### 3.1. Characterizations of hiPSC-CMs

To examine the gene expression patterns in hiPSCs and hiPSC-CMs, quantitative qPCR analysis was carried out at the beginning (day 0) and at different time points after the onset of differentiation. The results showed that the pluripotency gene POU5F1 (POU class 5 homeobox 1) decreased, while the typical cardiac genes, TNNT2 (troponin T type 2), MYH6 (myosin heavy chain 6), MYL2 (myosin regulatory light chain 2), NKX2.5 (NK2 homeobox 5), and ACTN2 (actinin alpha 2), increased over time (Figure S1).

**Figure 1:**
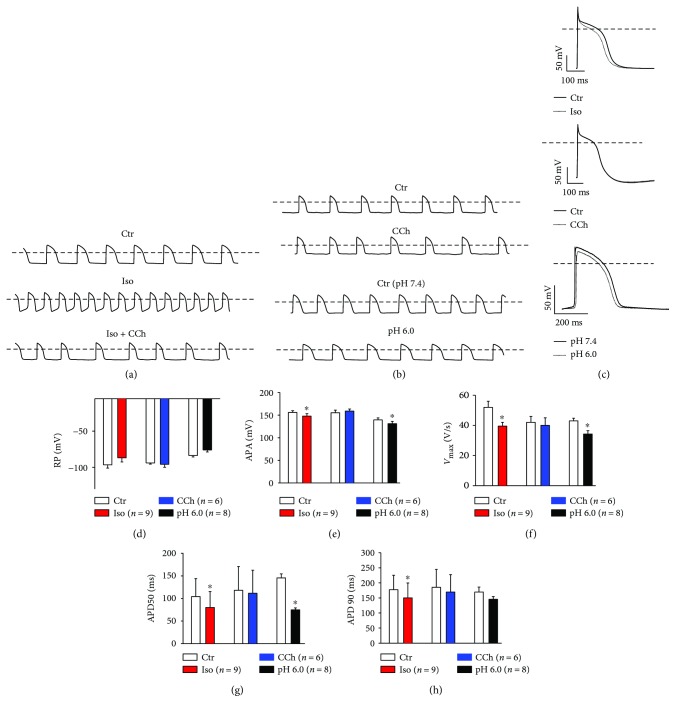
Effects of Iso and CCh as well as of acidosis on APs in hiPSC-CMs. (a) Representative spontaneous APs recorded before (Ctr) and after application of 10 *μ*M Iso and 10 *μ*M Iso + 30 *μ*M CCh. (b) Representative spontaneous APs recorded before (Ctr) and after application of 30 *μ*M CCh or extracellular solution with a pH value of 6.0. (c–h) APs were recorded at a fixed frequency of 1 Hz to analyze the changes of the AP parameters induced by Iso, CCh, and acidosis. Representative traces of APs (c), mean values of resting potential (d), AP amplitude (e), maximal depolarization speed (f), APD50 (g), and APD90 (h) are shown. Values given are mean ± SEM. *n*: number of cells. ^∗^ *p* < 0.05 versus Ctr.

Next, we checked the functional responses of hiPSC-CMs to some physiological factors like pH value and adrenergic and cholinergic stimulation. The spontaneous cell beating was accelerated by the application of 10 *μ*M isoprenaline (Iso). In contrast, a reduction of the pH in the perfused solution decreased the beating frequency. The effect of Iso could be reversed by 30 *μ*M carbachol (CCh) (Figure 1(a)). CCh alone, however, failed to change the cell beating frequency (Figure 1(b)). When hiPSC-CMs were stimulated at a fixed frequency of 1 Hz, Iso reduced *V*_max_ (the maximal depolarization velocity) of action potentials (APs) and shortened APD50 and APD90 (repolarization of AP at 50 and 90%) but did not influence the RP (resting potential) and APA (amplitude of AP). Acidosis reduced *V*_max_ and shortened APD50. CCh alone showed no effects on all the AP parameters (Figures 1(c)–1(h)).

**Figure 2:**
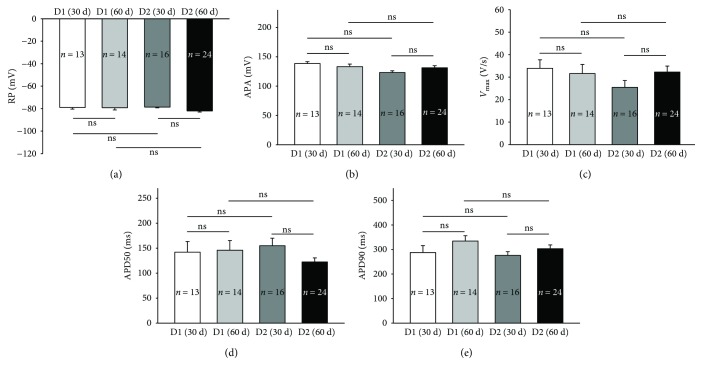
Comparison of action potential parameters in cells from different subjects and after different differentiation times. Action potentials were measured in cells from the two healthy donors (D1 and D2) 30 to 40 days (30 d) and 50 to 60 days (60 d) after the onset of differentiation. (a) Mean values of resting potential (RP). (b) AP amplitude (APA). (c) Maximal depolarization speed (*V*_max_). (d) AP duration at 50% repolarization (APD50). (e) AP duration at 90% repolarization (APD90). Values given are mean ± SEM. *n*: number of cells. ns: *p* > 0.05.

Then, we checked the possible variations of APs in cells measured at different time points or from different subjects. We observed very similar AP parameters in cells of two time groups (days 30 to 40 and days 50 to 60) and from the two donors (D1 and D2) (Figure 2).

### 3.2. Ion Channel and Receptor Expression in hiPSC-CMs

**Figure 3:**
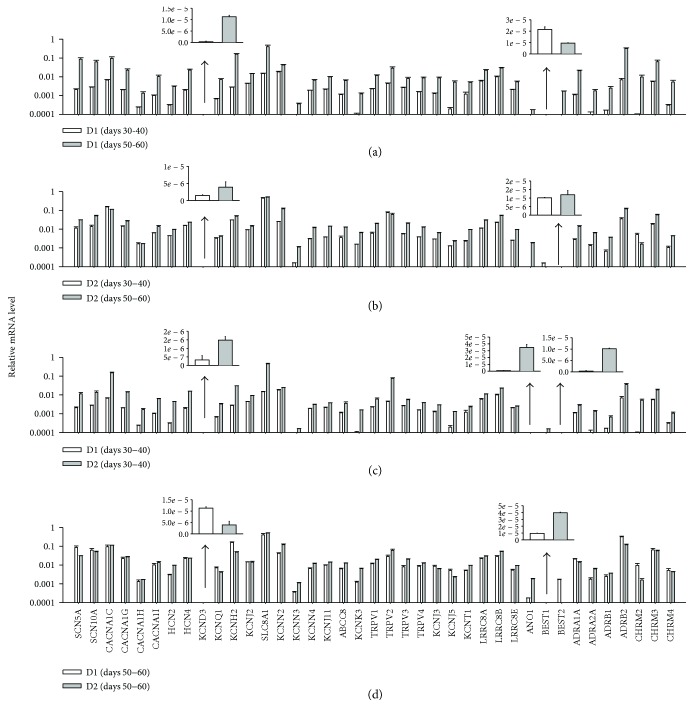
Ion channel and receptor expression in hiPSC-CMs. Relative mRNA levels (normalized with GAPDH) of different ion channels and adrenoceptors as well as of muscarinic receptors were analyzed by qPCR in hiPSC-CMs. (a) Mean values of relative mRNA expression of ion channels and receptors in cells from donor 1 (D1) 30 to 40 days (days 30–40) and 50 to 60 day (days 50–60) after the onset of differentiation. (b) Mean values of relative mRNA expression of ion channels and receptors in cells from donor 2 (D2) 30 to 40 days (days 30–40) and 50 to 60 days (days 50–60) after the onset of differentiation. (c) Mean values of relative mRNA expression of ion channels in cells from donor 1 (D1) and donor 2 (D2) 30 to 40 days after the onset of differentiation. (d) Mean values of relative mRNA expression of ion channels in cells from donor 1 (D1) and donor 2 (D2) 50 to 60 days after the onset of differentiation. Results shown are mean ± SEM.

To examine the ion channel expression profile in hiPSC-CMs, the mRNA expression level in cells with different differentiation time points (day 30 and day 60) or from different subjects (D1 and D2) was analyzed by qPCR (Figure 3). The expression of SCN5A (Na^+^ channel, Nav1.5); SCN10A (Na^+^ channel, Nav1.8); CACNA1C (L-type Ca^2+^ channel); CACNA1G (T-type Ca^2+^ channel, Cav3.1); CACNA1H (T-type Ca^2+^ channel, Cav3.2); CACNA1I (T-type Ca^2+^ channel, Cav3.3); SLC8A1 (Na^+^/Ca^2+^-exchanger, NCX1); HCN2 and HCN4 (I_f_ channel); KCND3 (I_to_, Kv4.3); KCNH2 (I_Kr_, Kv11.1); KCNQ1 (I_Ks_, Kv7.1); KCNJ2 (Kir2.1, I_K1_); KCNJ11 (K_ATP_, alpha-subunit); ABCC8 (K_ATP_, beta-subunit SUR1); KCNN2 (SK2); KCNN3 (SK3); KCNN4 (SK4); KCNK3 (TASK-1); KCNT1(Slo2.2, Na^+^-activated K^+^ channel); KCNJ3 (GIRK1, KACh), KCNJ5 (GIRK4, KACh); LRRC8A, LRRC8B, and LRRC8E (volume-regulated chloride channels); ANO1, BEST1, and BEST2 (calcium-activated chloride channels); and TRPV1, TRPV2, TRPV3, and TRPV4 which are known to exist in native cardiomyocytes was also detected in hiPSC-CMs.

We also checked the expression of adrenergic and muscarinic receptors in hiPSC-CMs. The gene expression of four adrenoceptors, alpha 1(ADRA1A), alpha 2 (ADRA2A), beta 1 (ADRB1), and beta 2 (ADRB2), as well as of three muscarinic receptors, M2 (CHRM2), M3 (CHRM3), and M4 (CHRM4), was detected with high expression of beta 2 (ADRB2) and M3 (CHRM3) (Figure 3).

### 3.3. Ion Channel Currents in hiPSC-CMs

**Figure 4:**
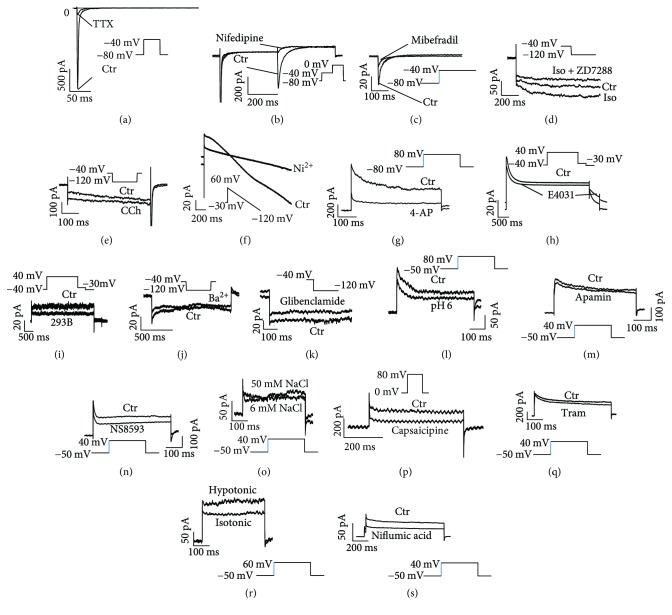
Ion channel currents in hiPSC-CMs. Whole currents were evoked by the indicated protocols, respectively. Specific blockers or solutions were applied to confirm the identity of expected currents. (a) Representative traces of TTX- (20 *μ*M) sensitive peak I_Na_. (b) Representative traces of nifedipine- (1 *μ*M) sensitive I_Ca-L_. (c) Representative traces of mibefradil- (10 *μ*M) sensitive I_Ca-T_. (d) Representative traces of ZD7288- (30 *μ*M) sensitive I_f_. (e) Representative traces of CCh- (10 *μ*M) activated I_KACh_. (f) Representative traces of Ni^2+^- (5 mM) sensitive I_NCX_. (g) Representative traces of 4-AP- (3 mM) sensitive I_to_. (h) Representative traces of E-4031- (3 *μ*M) sensitive I_Kr_. (i) Representative traces of chromalol 293B- (10 *μ*M) sensitive I_Ks_. (j) Representative traces of Ba^2+^- (100 *μ*M) sensitive I_K1_. (k) Representative traces of glibenclamide- (10 *μ*M) sensitive I_KATP_. (l) Representative traces of pH- (6.0) sensitive potassium channel current. (m) Representative traces of apamin- (100 nM) sensitive I_SK1–3_. (n) Representative traces of NS8593- (10 *μ*M) sensitive I_SK1–3_. (o) Representative traces of Na^+^- (50 mM) sensitive I_KNa_. (p) Representative traces of capsaicipine- (100 *μ*M) sensitive TRPV1 current. (q) Representative traces of TRAM 34- (100 nM) sensitive I_SK4._ (r) Representative traces of hypotonic solution- (220 mOsm/L) activated I_Cl-vol_. (s) Representative traces of niflumic acid- (100 *μ*M) sensitive I_Cl-Ca_.

To check whether the detected ion channels are functionally expressed in the cell membrane, we measured the individual ion channel currents with the help of special recording protocols, solutions, and channel blockers. The ion channel currents I_Na_, I_Ca-L_, I_Ca-T_, I_NCX_, I_f_ (funny current), I_KACh_ (acetylcholine-activated K^+^ current), I_to_, I_Kr_, I_Ks_, I_K1_(inward rectifier K^+^ channel current), I_SK1–3_, I_SK4_, I_KATP_, I_K-pH_ (pH-sensitive currents), I_Cl-vol_ (volume-regulated Cl^−^ channel current), I_Cl-Ca_ (Ca^2+^-activated Cl^−^ channel current), and TRPV1 channel currents could be measured, but I_f_ and I_K1_ were much smaller than those in native cardiomyocytes reported by previous studies. Na^+^-activated K^+^ currents (I_KNa_) were detected but very small in hiPSC-CMs (Table 1 and Figure 4). When the currents measured during 30 to 40 days after the onset of differentiation were compared with those recorded during 50 to 60 days, significant changes in I_K1_, I_SK4_, and I_Cl-vol_ were observed (Table 1 and Figure S2). No significant differences in the currents were detected in cells from the two subjects D1 and D2 (Figure S3).

To assess the channel gating kinetics in cells with different differentiation time points, the activation, inactivation, and recovery from inactivation of I_Na_, I_Ca-L_, and I_to_ were analyzed. No significant differences were detected between the two differentiation time groups (Figures S4–S6).

### 3.4. Contributions of Ion Channels to APs in hiPSC-CMs

**Figure 5:**
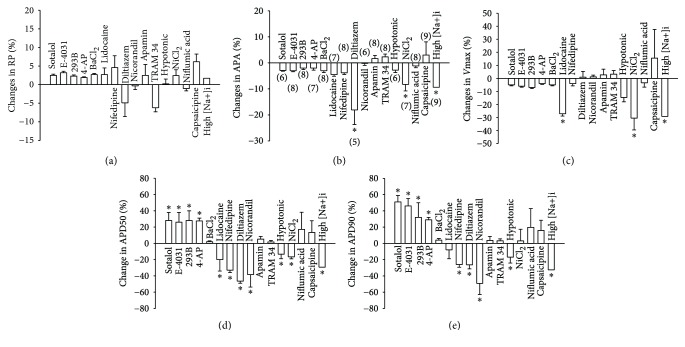
Influences of ion channels on APs in hiPSC-CMs. APs were recorded at 1 Hz. Specific ion channel blockers or activators were applied to assess the contribution of an ion channel to AP. Percentage changes induced by drugs were calculated as (*V*_drug_ − *V*_control_)/*V*_control_∗100, where *V*_control_ is the value before the application of a drug and *V*_drug_ is the value in the presence of a drug. (a) Percent (%) changes of resting potential (RP). (b) % changes of AP amplitude (APA). (c) % changes of maximal depolarization speed (*V*_max_). (d) % changes of APD at 50% repolarization (APD50). (e) % changes of APD at 90% repolarization (APD90). Values given are mean ± SEM. The number of cells (numbers in parentheses) is indicated in (b). ^∗^ *p* < 0.05 versus control.

To assess the functional roles of the individual ion channels, we tested the influence of different types of channels on APs when they were blocked or activated (Figure 5). No significant effect on the resting potential was observed (Figure 5(a)). Diltiazem (I_Ca-L_ blocker, 100 M), NiCl_2_ (I_NCX_ blocker, 5 mM), and high concentration (50 mM) of [Na^+^]_i_ (I_KNa_ activator) reduced AP amplitude (Figure 5(b)). Lidocaine, NiCl_2_, and high concentration of [Na^+^]_i_ reduced *V*_max_ (Figure 5(c)). When the frequency was fixed at 1 Hz, lidocaine, nifedipine, diltiazem, nicorandil (I_KATP_ activator), NiCl_2_, and the hypotonic (I_Cl-vol_ activator) and high [Na^+^]_i_ solution shortened APD50 and APD90. The I_to_ blocker 4-AP, I_Kr_ blockers E-4031 and sotalol, and I_Ks_ blocker chromalol 293B prolonged APD50 and APD90. The I_K1_ blocker BaCl_2_, I_SK_ blocker apamin, I_SK4_ blocker TRAM 34, I_Cl-Ca_ blocker niflumic acid, and TRPV1 blocker capsaicipine as well as K_ACh_ activator carbachol (CCh) did not significantly change APs (Figures 1 and 5).

### 3.5. Regulation of Ion Channels by Adrenergic and Cholinergic Stimulation

**Figure 6:**
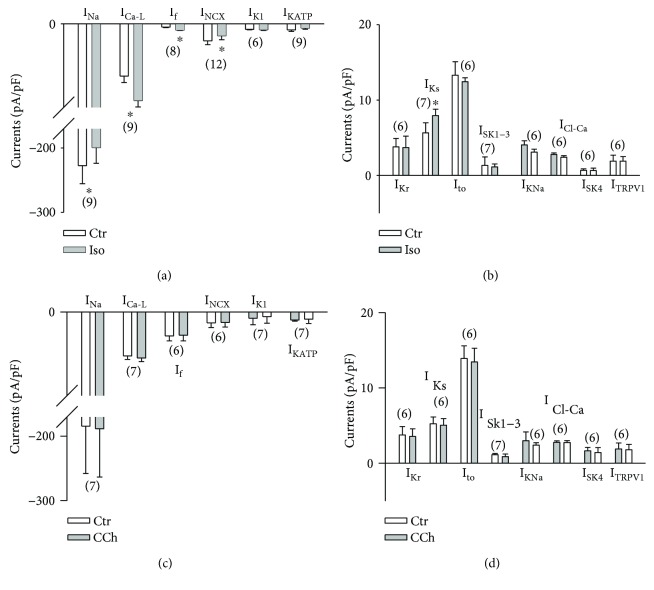
Effects of Iso and CCh on ion channels in hiPSC-CMs. Different ion channel currents were recorded under different conditions. Iso (10 *μ*M) or CCh (10 *μ*M) was applied to check the effect on the recorded currents. (a) Mean values of inward currents in the absence and presence of Iso. (b) Mean values of outward currents in the absence and presence of Iso. (c) Mean values of inward currents in the absence and presence of CCh. (d) Mean values of outward currents in the absence and presence of CCh. Values given are mean ± SEM. The numbers in parentheses indicate the number of cells. ^∗^ *p* < 0.05 versus control.

Finally, we investigated the responses of the ion channel to adrenergic and cholinergic stimulation. Application of 10 *μ*M Iso to hiPSC-CMs enhanced I_Ca-L_, I_f_, and I_Ks_ but reduced I_Na_ and I_NCX_ without an effect on I_K1_, I_KATP_, I_to_, I_Kr_, I_SK1–3_, I_K-Na_, I_Cl-Ca_, I_SK4_, and I_TRPV1_. No significant effect of CCh (10 *μ*M) on the currents was detected (Figure 6).

## 4. Discussion

In this study, we investigated the ion channel expression, function, and regulation by adrenergic and cholinergic stimulation in hiPSC-CMs. The novelties of this study are (1) detecting both the gene expression and currents of K_ACh_, K_Na_, Cl_Ca_, Cl_vol_, and TRPV channels in hiPSC-CMs; (2) examining the possible functional contributions to the action potential of I_SK1–3_, I_SK4_, K_Na_, I_Cl-Ca_, I_Cl-vol_, I_K-pH_, and I_TRPV1_; and (3) testing the effects of adrenergic and cholinergic stimulation on I_SK1–3_, I_SK4_, K_Na_, I_Cl-Ca_, I_TRPV1_, I_Na_, I_to_, I_NCX_, and I_KATP_.

We have successfully generated hiPS-CMs with the majority of cells showing ventricular-like APs [26]. The successful differentiation of hiPS cells into cardiomyocytes was confirmed by the decrease in expression of the pluripotency gene, the increase in cardiac genes (Figure S1), and the spontaneously cell beating.

To characterize the physiological property of hiPSC-CMs, we checked their responses to some physiological factors like pH value and adrenergic and cholinergic stimulation. Application of 10 *μ*M isoprenaline (Iso) accelerated the beating frequency of the cells. The effect of Iso could be reversed by 30 *μ*M carbachol (CCh) (Figure 1(a)). These data showed the similarity between hiPSC-CMs and native cardiomyocytes [28–30]. Acidosis reduced frequency and *V*_max_ (the maximal depolarization velocity) and shortened APD50 and APD90 (repolarization of AP at 50 and 90%) but did not influence the RP (resting potential) and APA (amplitude of AP) (Figures 1(c)–1(h)). To date, these effects of acidosis on AP parameters have not been shown in hiPSC-CMs.

The ion channel expression and functions in native ventricular cardiomyocytes have been well documented [31–33]. Major ion channels, like SCN5A, L-type Ca^2+^ channel, I_to_, I_Kr_, I_Ks_, and I_K1_, play critical roles in the electrical activities and functions of healthy and diseased cardiomyocytes. Some channels like K_ATP_, I_f_, SK_1–3_, and TASK-1 are associated with some heart diseases. Therefore, it is important to know the similarity and also the difference of ion channels between the hiPSC-CMs and the native cardiomyocytes. To get the information about ion channel expression, function, and regulation in hiPSC-CMs, we employed qPCR and patch clamp techniques to study ion channels in our hiPSC-CMs from the two donors with different differentiation times.

First, we checked the major group, the most frequently investigated ion channels, such as the Na, Ca, transient outward current (I_to_), delayed rectifier K current (I_Kr_, I_Ks_), inward rectifier current (I_K1_), Na/Ca exchanger (I_NCX_), and funny current (I_f_) channels. Although those channels have been investigated in hiPSC-CMs [9, 34, 35], at least two rationales led us to check them in our hiPSC-CMs: (1) cell lines generated with different protocols may show different properties including ion channel expression and functions and (2) some of those channels were not investigated in detail and some important data like the regulation of some channels by adrenergic and cholinergic stimulation are still lacking. In our recent study, we investigated the effects of LPS on I_Na_, I_Ca-L_, I_to_, I_Kr_, I_Ks_, I_NCX_, I_SK1–3_, I_K-pH_, and I_KATP_ [27]. But we did not examine their functional effects on action potentials. The effects of adrenergic and cholinergic stimulation on those channels were not checked either. In the current study, we focused on their functional contributions to AP and regulations by adrenergic and cholinergic stimulation. Indeed, we have added some new data. For example, we observed a reduction of I_Na_ induced by Iso, which was not shown in hiPSC-CMs and different from some reported data in native cardiomyocytes showing an increase in I_Na_ by Iso [36]. Furthermore, we observed an inhibition of I_NCX_ by Iso, which has not been shown at least in hiPSC-CMs so far.

Next, we assessed some ion channels that have not been investigated in detail or have not been reported in hiPSC-CMs but have been shown to exist in native cardiomyocytes.

The functional T-type calcium channel exists in the conduction system of human heart but not in ventricular myocytes under physiological condition [37]. However, they can be expressed in atrial or ventricular myocytes in some diseases like heart failure [37]. One study reported the existence of T-type calcium channels in hiPSC-CMs by showing the currents evoked by a pulse from −90 to −40 mV [38]. But no clear evidence was provided in that study. In the current study, we detected the mRNA of the three isoforms of the T-type calcium channel CACNA1G (T-type Ca^2+^ channel, Cav3.1), CACNA1H (T-type Ca^2+^ channel, Cav3.2), and CACNA1I (T-type Ca^2+^ channel, Cav3.3). In addition, we measured the Ca^2+^ current in Na^+^-free solution plus 20 *μ*M TTX, which is sensitive to mibefradil, a T-type calcium channel blocker. Therefore, the current study provides clearer evidences for the presence of T-type calcium channels in hiPSC-CMs (Figures 3 and 4(c) and Table 1).

The acetylcholine-activated potassium (K_ACh_) channel is involved in cholinergic regulation of the heart rate. Although the atrial-selective ion current I_KACh_ has recently been reported in human embryonic stem cell-derived atrial-like CMs [39], I_KACh_ has not been studied in hiPSC-CMs. Therefore, we checked them in our cell line. Two isoforms of I_KACh_ channels KCNJ3 and KCNJ5 (Kir3.1 and Kir3.4) could be detected by qPCR (Figure 3). Patch clamp measurements displayed carbachol-activated K^+^ currents (Figure 4(e) and Table 1), suggesting expression of functional K_ACh_ channels.

The sodium-activated potassium (K_Na_) channel has been shown to be present in cardiomyocytes from different species [40, 41]. The K_Na_ channel can only be activated by high concentration of intracellular Na^+^, which cannot be reached under physiological conditions. Therefore, the physiological meaning of K_Na_ channels is not clear. The molecular identity of K_Na_ channels in cardiomyocytes is not clear either. Whether this type of channels exists in hiPSC-CMs is not known. We detected in our hiPSC-CMs the presence of the KCNT1 (Slo2.2) channel, a K_Na_ channel that is widely expressed in the brain. But K_Na_ currents are very small (Figure 4(o) and Table 1), suggesting a minor contribution to the electrical activity of hiPSC-CMs.

Various types of Cl^−^ currents have been reported in cardiomyocytes from different regions of the heart and in different species, including the stretch- or volume-activated Cl^−^ current (I_Cl-vol_) and the Ca^2+^-activated Cl^−^ current (I_Cl-Ca_) [42, 43]. Whether these channels exist in hiPSC-CMs is unknown. In current study, both the PCR analysis and patch clamp measurements exhibited the functional expression of the volume- and Ca^2+^-activated chloride channels in hiPSC-CMs (Table 1 and Figures 3 and 4(r) and 4(s)).

Transient receptor potential (TRP) channels are a big family of ion channels, which have been reported to exist in the heart [44]. To our knowledge, functional TRP channels have not been reported in hiPSC-CMs. In this study, we found out the expression of TRPV1–4 at mRNA level (Figure 3) and the presence of capsaicipine- (TRPV1 blocker) sensitive currents in hiPSC-CMs, suggesting that at least TRPV1 is functionally expressed. Because of the limitation-specific blocker, we could not identify the currents conducted by TRPV2–4 channels.

APs in cardiomyocytes are critical for normal cell function, especially the normal rhythm. Any changes in APs may be pathogenic. Although some ion channels, especially the major ion channels, have been assessed in hiPSC-CMs, the contributions of many channels to action potentials have not been examined yet. To further check the physiological and pathological importance of the detected ion channels in hiPSC-CMs, we investigated their influence on APs (Figure 5). No significant effects of the tested channel blockers or activators on RP were observed (Figure 5(a)). It is known that I_K1_, which is an important determinant of RP in native cardiomyocytes, is small in hiPSC-CMs. The RP in hiPSC-CMs should be determined by some other ion channel currents. One study demonstrated that I_Kr_ is the main determinant [45]. Our data suggest that the resting potential is probably determined by multiple ion channels. Thus, the inhibition or activation of only one channel has a negligible effect on RP. The I_Ca-L_ blocker diltiazem and I_NCX_ blocker NiCl_2_ reduced significantly APA (Figure 5(b)), suggesting that I_Ca-L_ and I_NCX_ contributed to the amplitude of APs. The I_Na_ blocker lidocaine reduced *V*_max_ significantly (Figure 5(c)), indicating that I_Na_ determined the depolarization speed. NiCl_2_ and high [Na^+^]_i_ also reduced *V*_max_, probably due to their effects on I_Na_. Potassium channel blockers (4-AP, sotalol, E-4031, and chromalol 293B) prolonged APD, whereas, lidocaine, nifedipine, diltiazem, NiCl_2_, and the K_ATP_ activator nicorandil shortened APD. All these data confirmed the expected contributions of I_Na_, I_Ca-L_, I_to_, I_Kr_, I_Ks_, I_NCX_, and I_KATP_ to the repolarization of APs in hiPSC-CMs and are indicative of the similarity between hiPSC-CMs and native cardiomyocytes. Lidocaine and NiCl_2_ shortened APD50 more strongly than they did on APD90, suggesting minor effects of I_Na_ and I_NCX_ on the late phase of APD. In addition, hypotonic solution and high [Na^+^]_i_ shortened APD, meaning that I_Cl-vol_ and I_KNa_ may influence the repolarization. But their effects on other ion channels cannot be excluded due to lack of specific blockers for both channels. Furthermore, we found that the SK1–3 channel blocker apamin, SK4 channel blocker TRAM34, I_K1_ blocker BaCl_2_, I_Cl-Ca_ blocker niflumic acid, and TRPV1 channel blocker capsaicipine showed no significant effects on AP parameters, although the respective currents could be recorded. These data may hint at a minor contribution of the respective currents to APs in hiPSC-CMs. Acidosis reduced *V*_max_ and shortened APD50 significantly (Figures 1(f) and 1(g)) probably due to inhibition of inward currents or enhancement of some outward currents but not through the detected pH-sensitive TASK-1 channel currents because the reduction of I_TASK-1_ should prolong APD.

Adrenergic and cholinergic regulation is of importance for normal heart function. In some studies with hiPSC-CMs, the effects of isoprenaline (changes in beating frequency and APD) were checked and responses similar to those in native cardiomyocytes were shown [8]. Adrenalin has been shown to enhance I_Ks_ in hiPSC-CMs [8]. Iso enhanced I_Ca-L_ in hiPSC-CMs [38]. Both are in agreement with the reported effect in native cardiomyocytes [33]. But the regulation of many other ion channels by adrenergic and cholinergic stimulation has not been examined in hiPSC-CMs. We checked the responses of the detected ion channels to both adrenergic and cholinergic stimulation. We observed that Iso increased I_Ca-L_, I_f_, and I_Ks_, similar to the reported effects of Iso in native myocytes. Iso reduced I_Na_ in our hiPSC-CMs (Figure 6); this is different from its effect on I_Na_ in native cardiomyocytes [36]. Furthermore, we found that Iso showed no effects on I_SK1–3_, I_SK4_, K_ATP_, I_KNa_, I_Cl-Ca_, and I_Cl-vol_ but reduced I_NCX_ (Figure 6), which are different from the reported data in native cells. CCh has been shown to reduce the beating frequency of hiPSC-CMs [46]. This suggests possible effects of CCh on ion channels, which have not been tested in hiPSC-CMs. In the current study, the effects of CCh on different channel currents were examined. Although no direct effect of CCh on all the tested channel currents and no effect of Iso on most of the tested currents were detected, those data may also provide relevant information for future studies with respect to ion channels in hiPSC-CMs.

This and previous studies demonstrated clearly that hiPSC-CMs responded to adrenergic and cholinergic stimulation. Transcriptome analysis revealed the expression of adrenoceptors in iPS cell-derived cardiomyocytes [47, 48]. In the normal human heart, beta 1 adrenoceptors predominate over beta 2 with the ratio being approximately 4 : 1 [49, 50]. Our data showed the presence of alpha 1, alpha 2, beta 1, and beta 2, and the mRNA level of beta 2 is higher than that of beta 1 in hiPSC-CMs (Figure 3), different from native human cardiomyocytes. Regarding muscarinic receptors, the predominant cardiac and ventricular isoform is M2. However, there are indications that other family members might be present, in particular M1 and M3 receptors [51]. We detected M2, M3, and M4 with M3 having the highest expression (Figure 3), again different from native cardiomyocytes.

It has been shown that the AP morphology and some ion channel currents varied with cell ages (differentiation times) [38, 52, 53]. We checked the AP parameters and some ion channel currents in cells measured during 30 to 40 days and 50 to 60 days after the start of differentiation. Although PCR data showed an increase in most ion channels, patch clamp measurements showed that only some channel currents like I_Na_, I_SK4_, and I_Cl-vol_ were significantly changed. More importantly, AP parameters exhibited no time-dependent changes. In addition, we observed similar APs and ion channel currents in cells from both healthy donors, especially in older cells (Figures 2 and S3). Even though only two cell lines (one from each donor) were investigated in this study and the variation of ion channels in different cell lines cannot be excluded, the AP and current data from the two donors showed that the variation between D1 and D2 is not large. The qPCR data of some ion channels did not correlate with the current data. One reason could be that the gene of an ion channel was transcribed, but there was no equivalent protein in the membrane. Another reason could be that the channels were in the membrane, but the channel open probability was changed by some intracellular factors.

Our data displayed that hiPSC-CMs possess similarities but also differences when compared with the reported data from mature cardiomyocytes. However, quantitative comparison should be performed with caution. The reported data varied in different research groups, and more importantly, different groups performed the studies under different experimental conditions like temperatures, solutions, recording protocols, and tissues species. It would be more plausible to compare the data from hiPSC-CMs and native human cardiomyocytes under the same experimental conditions. Due to limited availability of human ventricular cardiomyocytes, we could not compare our data from hiPSC-CMs with that from the native human cardiomyocytes under the same condition. Nevertheless, this study added some novel data about the functional ion channels and their regulation by adrenergic and cholinergic stimulation in hiPSC-CMs.

## 5. Conclusions

This study demonstrated that the ion channels that exist in mature cardiomyocytes are also present in hiPSC-CMs with similarities or differences in some aspects. The novel data from this study may provide useful information for future studies regarding ion channels in hiPSC-CMs.

## Figures and Tables

**Table 1:** Ion channel currents in hiPSC-CMs (D1).

Channel current		Ctr (pA/pF)	Blocker (pA/pF)	Sensitive current (pA/pF)	At mV	*n*
I_Na_	(Days 30–40)	−30 ± 27.6	−5.9 ± 5.5	−23.7 ± 22.1	−40	7
	(Days 50–60)	−52.8 ± 44.0	−10.5 ± 3.6	−42.3 ± 35.1	−40	6

I_Ca-L_	(Days 30–40)	−9.7 ± 1.1	−3.2 ± 0.6	−6.4 ± 1.2	10	10
	(Days 50–60)	−13.2 ± 3.8	−4.4 ± 1.2	−8.8 ± 2.5	10	10

I_Ca-T_	(Days 30–40)	−2.1 ± 0.8	−1.0 ± 0.6	−1.1 ± 0.3	−30	7

I_f_	(Days 30–40)	−7.5 ± 0.9	−6.4 ± 0.9	−1.2 ± 0.3	−120	8
	(Days 50–60)	−3.4 ± 0.8	−1.8 ± 0.4	−1.6 ± 0.5	−120	5

I_NCX_	(Days 30–40)	−6.1 ± 1.0	−3.7 ± 0.8	−2.4 ± 0.6	−85	9
	(Days 50–60)	−9.6 ± 1.5	−5.5 ± 1.0	−4.2 ± 0.8	−85	15

I_K1_	(Days 30–40)	−13.3 ± 4.0	−12.5 ± 4.0	−0.7 ± 0.2	−120	7
	(Days 50–60)	−13.4 ± 0.9	−11.5 ± 0.9	−1.9 ± 0.4^∗^	−120	8

I_to_	(Days 30–40)	13.0 ± 2.1	6.9 ± 1.5	6.1 ± 1.0	70	14
	(Days 50–60)	10.7 ± 2.1	5.7 ± 1.3	5.0 ± 0.9	70	10

I_Kr_	(Days 30–40)	2.6 ± 0.4	1.8 ± 0.3	0.9 ± 0.2	40	8
	(Days 50–60)	2.5 ± 0.4	1.6 ± 0.3	1.0 ± 0.2	40	7

I_Ks_	(Days 30–40)	1.7 ± 0.5	0.9 ± 0.3	0.8 ± 0.5	40	15
	(Days 50–60)	1.3 ± 0.4	0.5 ± 0.2	0.8 ± 0.3	40	10

I_KATP_	(Days 30–40)	−4.1 ± 0.4	−2.2 ± 0.8	−1.9 ± 0.4	−120	5
	(Days 50–60)	−4.7 ± 0.9	−2.4 ± 0.8	−2.2 ± 0.4	−120	5

I_KNa_	(Days 30–40)	2.2 ± 0.2^a^	2.1 ± 0.2^b^	0.1 ± 0.1	60	8

I_K-pH_	(Days 30–40)	7.0 ± 2.6^c^	5.1 ± 2.8^d^	1.8 ± 0.8	60	5
	(Days 50–60)	12.7 ± 3.9^c^	9.3 ± 3.0^d^	3.4 ± 2.1	60	4

S_K1–3_	(Days 30–40)	1.9 ± 0.7	1.5 ± 0.5	0.5 ± 0.4	40	10
	(Days 50–60)	3.1 ± 1.5	2.5 ± 1.2	0.6 ± 0.3	40	7

I_KACh_	(Days 30–40)	−6.6 ± 0.3	−4.09 ± 0.6	−2.6 ± 0.6	−120	6
	(Days 50–60)	−6.7 ± 0.3	−4.5 ± 0.3	−2.2 ± 0.3	−120	6

I_TRPV1_	(Days 30–40)	3.8 ± 1.4	2.4 ± 1.1	1.4 ± 0.5	40	5
	(Days 50–60)	3.5 ± 1.4	2.7 ± 1.1	0.8 ± 0.4	40	5

I_Cl-Ca2_	(Days 30–40)	4.3 ± 1.3	3.7 ± 0.8	0.6 ± 0.8	50	6
	(Days 50–60)	4.7 ± 1.2	4.0 ± 0.9	0.7 ± 0.4	50	7

I_SK4_	(Days 30–40)	5.8 ± 0.8	3.0 ± 0.7	2.8 ± 0.4	50	5
	(Days 50–60)	2.6 ± 0.6	1.5 ± 0.3	1.1 ± 0.9^∗^	50	5

I_Cl-vol_	(Days 30–40)	0.8 ± 0.2^e^	1.1 ± 0.5^f^	0.3 ± 0.5	60	6
	(Days 50–60)	0.9 ± 0.2^e^	2.2 ± 0.6^f^	1.2 ± 0.6^∗^	60	6

^∗^ *p* < 0.05 versus days 30–40; ^a^[Na+]i = 6 mM; ^b^[Na+]i = 50 mM; ^c^pH = 7.4; ^d^pH = 6.0; ^e^isotonic solution; ^f^hypotonic solution.

## Supplementary Materials

Supplementary 1 Table S1: list of genes, RefSeq numbers, and primers for qPCR.

Supplementary 2 Figure S1: characterizations of hiPSC-CMs. The qPCR analysis was carried out to assess the relative mRNA expression (normalized with GAPDH) of the pluripotency gene POU5F (A) and the cardiac genes MLY2 (B), TNNT2 (C), NKX2.5 (D), ACTN2 (E), and MYH6 (F) at different times after the onset of differentiation. Results shown are mean ± SEM. ^∗^ *p* < 0.05 versus d0.

Supplementary 3 Figure S2: comparison of ion channel currents in cells after different differentiation times. Shown are I–V curves of ion channel currents in cells from donor 1 (D1) 30 to 40 days (30 d) and 50 to 60 days (60 d) after the onset of differentiation. (A) Peak Na channel currents (I_Na_). (B) Peak L-type Ca channel currents (I_Ca-L_). (C) Peak transient outward K channel currents (I_to_). (D) Steady-state rapidly delayed rectifier K currents (I_Kr_). (E) Steady-state slowly delayed rectifier K currents (I_Ks_). (F) Na/Ca exchanger currents (I_NCX_). (G) Funny currents (I_f_). (H) Inward rectifier K currents (I_K1_). (I) Small-conductance Ca-activated K currents (I_SK1–3_). (J) Intermediate-conductance Ca-activated K currents (I_SK4_). (K) pH-sensitive K currents (I_K-pH_). (L) ATP-sensitive K current (I_KATP_). (M) Transient receptor potential type V1 current (I_TRPV1_). (N) Ca-activated Cl current (I_Cl-Ca_). (O) Volume-regulated Cl current (I_Cl-vol_). Values given are mean ± SEM. ^∗^ *p* < 0.05.

Supplementary 4 Figure S3: comparison of ion channel currents in cells from different donors. Shown are ion channel currents in cells from donor 1 (D1) and donor 2 (D2) 50 to 60 days after the onset of differentiation. (A) Peak Na channel currents (I_Na_). (B) Peak L-type Ca channel currents (I_Ca-L_). (C) Peak transient outward K channel currents (I_to_). (D) Steady-state rapidly delayed rectifier K currents (I_Kr_). (E) Steady-state slowly delayed rectifier K currents (I_Ks_). (F) Inward rectifier K currents (I_K1_). (G) Na/Ca exchanger currents (I_NCX_). (H) ATP-sensitive K current (I_KATP_). (I) Small-conductance Ca-activated K currents (I_SK1–3_). (J) Intermediate-conductance Ca-activated K currents (I_SK4_). (K) Ca-activated Cl current (I_Cl-Ca_).

Supplementary 5 Figure S4: comparison of Na channel kinetics in cells after different differentiation times. (A) Activation curves of peak Na channel currents (I_Na_). (B) Inactivation curves of peak I_Na_. (C) Recovery from inactivation of peak I_Na_. (D) Half maximal voltage of the activation of I_Na_. (E) Half maximal voltage of the inactivation of I_Na_. (F) Time constants (tau) of the recovery of I_Na_.

Supplementary 6 Figure S5: comparison of L-type Ca channel kinetics in cells after different differentiation times. (A) Activation curves of peak Ca channel currents (I_Ca-L_). (B) Inactivation curves of peak I_Ca-L_. (C) Recovery from the inactivation of peak I_Ca-L_. (D) Half maximal voltage of the activation of I_Ca-L_. (E) Half maximal voltage of the inactivation of I_Ca-L_. (F) Time constants (tau) of the recovery of I_Ca-L_.

Supplementary 7 Figure S6: comparison of kinetics of transient outward K channels in cells after different differentiation times. (A) Activation curves of peak transient outward K channel currents (I_to_). (B) Inactivation curves of peak I_to_. (C) Recovery from the inactivation of peak I_to_. (D) Half maximal voltage of the activation of I_to_. (E) Half maximal voltage of the activation of I_to_. (F) Time constants (tau) of the recovery of I_to_.
