# Supplementary material for: Ion Channel Expression and Characterization in Human Induced Pluripotent Stem Cell-Derived Cardiomyocytes
Source: Stem Cells Int. 2018 Jan 8;2018:6067096. doi: 10.1155/2018/6067096 (PMC5835237; doi:10.1155/2018/6067096)
Supplement: Supplementary 1 — Table S1: list of genes, RefSeq numbers, and primers for qPCR. [file 6067096.f1.docx]

**Ion channel expression and characterization in human-induced pluripotent stem cell-derived cardiomyocytes**

**Running title:** Ion channels in hiPSC-CMs

Zhihan Zhao^1,2*^, Huan Lan^1,2,3*^, Ibrahim El-Battrawy^1,2^, Xin Li^1^, Fanis Buljubasic^1,2^, Katherine Sattler^1,2^, Gökhan Yücel^1,2^, Siegfried Lang^1,2^, Malte Tiburcy^2,4^, Wolfram-Hubertus Zimmermann^,2,4^, Lukas Cyganek^2,5^, Jochen Utikal^2,6^, Thomas Wieland^2,7^, Martin Borggrefe^1,2^, Xiao-Bo Zhou^1,2,3^, Ibrahim Akin^1,2^

^1^First Department of Medicine, Faculty of Medicine, University Medical Centre Mannheim (UMM), University of Heidelberg, Mannheim, Germany

^2^DZHK (German Center for Cardiovascular Research), Partner Sites, Heidelberg-Mannheim and Göttingen, Germany

**^3^Key Laboratory of Medical Electrophysiology of Ministry of Education,** Institute of Cardiovascular Research, Southwest Medical University, Luzhou, Sichuan, China

^4^Institute of Pharmacology and Toxicology, University of Göttingen, Göttingen, Germany

^5^Stem Cell Unit, Clinic for Cardiology and Pneumology, University Medical Center Göttingen, Göttingen, Germany

^6^Skin Cancer Unit, German Cancer Research Center (DKFZ), Heidelberg and Department of Dermatology, Venereology and Allergology, University Medical Center Mannheim, University of Heidelberg, Mannheim, Germany;

^7^Institute of Experimental and Clinical Pharmacology and Toxicology, Medical Faculty Mannheim, University of Heidelberg, Mannheim, Germany

*equally contributed

**Address for correspondence**:

Xiao-Bo Zhou, MD

First Department of Medicine

University Medical Centre Mannheim

Theodor-Kutzer-Ufer 1-3

68167 Mannheim

Germany

**E-mail**: [Xiaobo.zhou@medma.uni-heidelberg.de](mailto:Xiaobo.zhou@medma.uni-heidelberg.de)

**Conflict of Interest: none**

**Supplemental data**

**Figure S1 Characterizations of hiPSC-CMs.** The qPCR analysis was carried out to assess the relative mRNA expression (normalized with GAPDH) of the pluripotency gene POU5F (A) and the cardiac genes MLY2 (B), TNNT2 (C), NKX2.5 (D), ACTN2 (E) and MYH6 (F) at different times after onset of differentiation. Results shown are mean ± SEM. *, p<0.05 vs. d0.

**Figure S2 Comparison of ion channel currents in cells after different differentiation times**. Shown are I-V curves of ion channel currents in cells from donor 1 (D1) 30 to 40 days (30d) and 50 to 60 days (60d) after onset of differentiation. A, peak Na channel currents (I_Na_). B, peak L-type Ca channel currents(I_Ca-L_). C, peak transient outward K channel currents (I_to_). D, steady state rapidly delayed rectifier K currents (I_Kr_), E, steady state slowly delayed rectifier K currents (I_Ks_). F, Na/Ca exchanger currents (I_NCX_). G, funny currents (I_f_). H, inward rectifier K currents (I_K1_). I, small conductance Ca-activated K currents (I_SK1-3_). J, intermediated conductance Ca-activated K currents (I_SK4_). K, pH-sensitive K currents (I_K-pH_). L, ATP-sensitive K current (I_KATP_). M, transient receptor potential type V1 current (I_TRPV1_). N, Ca-activated Cl current (I_Cl-Ca_). O, volume-regulated Cl current (I_Cl-vol_). Values given are mean ± SEM. * p<0.05.

**Figure S3 Comparison of ion channel currents in cells from different donors**. Shown are ion channel currents in cells from donor 1 (D1) and donor 2 (D2) 50 to 60 days after onset of differentiation. A, peak Na channel currents (I_Na_). B, peak L-type Ca channel currents (I_Ca-L_). C, peak transient outward K channel currents (I_to_). D, steady state rapidly delayed rectifier K currents (I_Kr_), E, steady state slowly delayed rectifier K currents (I_Ks_). F, inward rectifier K currents (I_K1_).. G, Na/Ca exchanger currents (I_NCX_). H, ATP-sensitive K current (I_KATP_). I, small conductance Ca-activated K currents (I_SK1-3_). J, intermediate conductance Ca-activated K currents (I_SK4_). K, Ca-activated Cl current (I_Cl-Ca_).

**Figure S4 Comparison of Na channel kinetics in cells after different differentiation times**. A, activation curves of peak Na channel currents (I_Na_). B, inactivation curves of peak I_Na_. C, recovery from inactivation of peak I_Na_. D, half maximal voltage of activation of I_Na_. E, half maximal voltage of inactivation of I_Na_. F, time constants (Tau) of recovery of I_Na_.

**Figure S5 Comparison of L-type Ca channel kinetics in cells after different differentiation times**. A, activation curves of peak Ca channel currents (I_Ca-L_). B, inactivation curves of peak I_Ca-L_. C, recovery from inactivation of peak I_Ca-L_. D, half maximal voltage of activation of I_Ca-L_. E, half maximal voltage of inactivation of I_Ca-L_. F, time constants (Tau) of recovery of I_Ca-L_.

**Figure S6 Comparison of kinetics of transient outward K channels in cells after different differentiation times**. A, activation curves of peak transient outward K channel currents (I_to_). B, inactivation curves of peak I_to_. C, recovery from inactivation of peak I_to_. D, half maximal voltage of activation of I_to_. E, half maximal voltage of activation of I_to_. F, time constants (Tau) of recovery of I_to_.
